# Whole-genome resequencing reveals genetic diversity, differentiation, and selection signatures of yak breeds/populations in Qinghai, China

**DOI:** 10.3389/fgene.2022.1034094

**Published:** 2023-01-10

**Authors:** Guangzhen Li, Jing Luo, Fuwen Wang, Donghui Xu, Zulfiqar Ahmed, Shengmei Chen, Ruizhe Li, Zhijie Ma

**Affiliations:** ^1^ Academy of Animal Science and Veterinary Medicine, Qinghai University, Xining, China; ^2^ Key Laboratory of Animal Genetics and Breeding on Tibetan Plateau, Ministry of Agriculture and Rural Affairs, Xining, China; ^3^ Plateau Livestock Genetic Resources Protection and Innovative Utilization Key Laboratory of Qinghai Province, Xining, China; ^4^ College of Animal Science and Technology, Northwest A&F University, Xianyang, China; ^5^ Faculty of Veterinary and Animal Sciences, University of Poonch Rawalakot, Rawalakot, Pakistan

**Keywords:** *Bos grunniens*, whole-genome resequencing, genomic diversity, population structure, selection signature

## Abstract

The Qinghai Province of China is located in the northeast region of the Qinghai–Tibetan Plateau (QTP) and carries abundant yak genetic resources. Previous investigations of archaeological records, mitochondrial DNA, and Y chromosomal markers have suggested that Qinghai was the major center of yak domestication. In the present study, we examined the genomic diversity, differentiation, and selection signatures of 113 Qinghai yak, including 42 newly sequenced Qinghai yak and 71 publicly available individuals, from nine yak breeds/populations (wild, Datong, Huanhu, Xueduo, Yushu, Qilian, Geermu, Tongde, and Huzhu white) using high-depth whole-genome resequencing data. We observed that most of Qinghai yak breeds/populations have abundant genomic diversity based on four genomic parameters (nucleotide diversity, inbreeding coefficients, linkage disequilibrium decay, and runs of homozygosity). Population genetic structure analysis showed that Qinghai yak have two lineages with two ancestral origins and that nine yak breeds/populations are clustered into three distinct groups of wild yak, Geermu yak, and seven other domestic yak breeds/populations. *F*_ST_ values showed moderate genetic differentiation between wild yak, Geermu yak, and the other Qinghai yak breeds/populations. Positive selection signals were detected in candidate genes associated with disease resistance (*CDK2AP2*, *PLEC*, and *CYB5B*), heat stress (*NFAT5*, *HSF1*, and *SLC25A48*), pigmentation (*MCAM*, *RNF26*, and *BOP1*), vision (*C1QTNF5*, *MFRP*, and *TAX1BP3*), milk quality (*OPLAH* and *GRINA*), neurodevelopment (*SUSD4*, *INSYN1*, and *PPP1CA*), and meat quality (*ZRANB1*), using the integrated PI, composite likelihood ratio (CLR), and *F*_ST_ methods. These findings offer new insights into the genetic mechanisms underlying target traits in yak and provide important information for understanding the genomic characteristics of yak breeds/populations in Qinghai.

## Introduction

The yak is a large, unique ungulate that lives in the climatically challenging conditions (limited oxygen, extreme cold, highly variable daytime and nighttime temperatures, and scanty flora) of the Qinghai–Tibetan Plateau (QTP) and nearby high-altitude regions (Wiener et al., 2003; Zhang et al., 2020). Domestic yak (*Bos grunniens*) is descended from wild yak (*Bos mutus*) (Qiu et al., 2015) and is an indispensable part of the Tibetan culture, providing basic resources such as meat, milk, transportation, fuels, and hides to Tibetans and other nomadic peoples living in high-altitude environments (Wiener et al., 2003; Jia et al., 2019; Jia et al., 2020). The Qinghai Province of China, located in the northeast region of the QTP, possesses a variety of yak genetic resources and is regarded as a major center of yak domestication (Guo et al., 2006; Ma, 2019). Due to its special geographical location, complex plateau climate, and long breeding history, Qinghai is home to some unique yak populations/breeds. For instance, two improved breeds (Datong and Ashdan) and four indigenous breeds (Qinghai Plateau, Huanhu, Xueduo, and Yushu) are currently recognized in this region (National Committee of Animal Genetic Resources, 2021).

According to our previous reports on genetic variations in Y-chromosomal markers, both wild and domestic yak in Qinghai have relatively high paternal genetic diversity with weak phylogeographic structures and two paternal origins (Ma et al., 2018; Ma et al., 2022). In addition, maternal genetic diversity of the wild and domestic yak in Qinghai indicates that wild and domestic yak have high levels of genetic diversity and can be clustered into three lineages (Wang et al., 2021; Li et al., 2022). Since a female domestic yak genome was first assembled in 2012, the domestic yak reference genome has been improved twice at the chromosomal level (Qiu et al., 2012; Ji et al., 2021; Zhang et al., 2021). Obviously, the completeness and accuracy of the latest yak reference genome (BosGru3.0, GCA_005887515.2) are significantly higher than those of the previously reported genomes (Zhang et al., 2021). This information has laid a strong foundation for further exploration of the genomic diversity, population structure, and phylogenetic relationships of yak breeds/populations at the genome level. Also, the availability of the high-quality yak reference genome, which was built using long-read sequencing technology (Zhang et al., 2021), has enabled the identification of the genetic basis of complex traits. Following the wide application of whole-genome sequencing (WGS), the genomic diversity, origin, domestication, population structure, coat color, and high-altitude adaptation of yak have become research hotspots (Qiu et al., 2012; Wang et al., 2014; Qiu et al., 2015; Liang et al., 2016; Zhang et al., 2016; Medugorac et al., 2017; Lan et al., 2018; Xie et al., 2018; E et al., 2019a; 2019b; Wang et al., 2019; Chai et al., 2020; Lan et al., 2021; Bao et al., 2022; Gao et al., 2022). Although a few Qinghai yak breeds/populations, including Qilian, Datong, Qinghai Plateau, and Huanhu, had been previously analyzed using WGS, this strategy could not comprehensively reveal genomic information on Qinghai yak breeds/populations because of its low-depth WGS data (average sequencing depth around ×7.2) and small number of samples (Chai et al., 2020). Additionally, the distribution area of some yak breeds such as Qinghai Plateau and Yushu are adjacent to the habitats of wild yak. Hybridization between domestic and wild yak is ubiquitous. Therefore, it is particularly necessary to explore the relationship between Qinghai domestic yak breeds/populations and wild yak using WGS data.

It is essential to thoroughly ascertain the genomic diversity, population structure, and selection signature of Qinghai yak breeds/populations to increase their potential breeding value. In this study, we performed high-depth WGS analysis of 113 yak, including 42 newly sequenced yak from nine Qinghai yak breeds/populations, to identify single-nucleotide polymorphisms (SNPs) based on the latest yak reference genome (BosGru3.0, GCA_005887515.2) and to reveal their genomic diversity and population structure, as well as candidate signatures of positive selection. The gathered information provides a baseline for the exploration and evaluation of yak breeds/populations in Qinghai, China. Moreover, it contributes to the conservation and utilization of these precious yak genetic resources.

## Materials and methods

### Ethical approval

This study was conducted following animal welfare requirements. The procedures approved for experiments were based on the recommendations of the Regulations for the Administration of Affairs Concerning Experimental Animals of China. The Institutional Animal Care and Use Committee of the Academy of Animal Science and Veterinary Medicine, Qinghai University, approved all the animal experiments in this study.

### Sample collection and whole-genome resequencing

**FIGURE 1:**
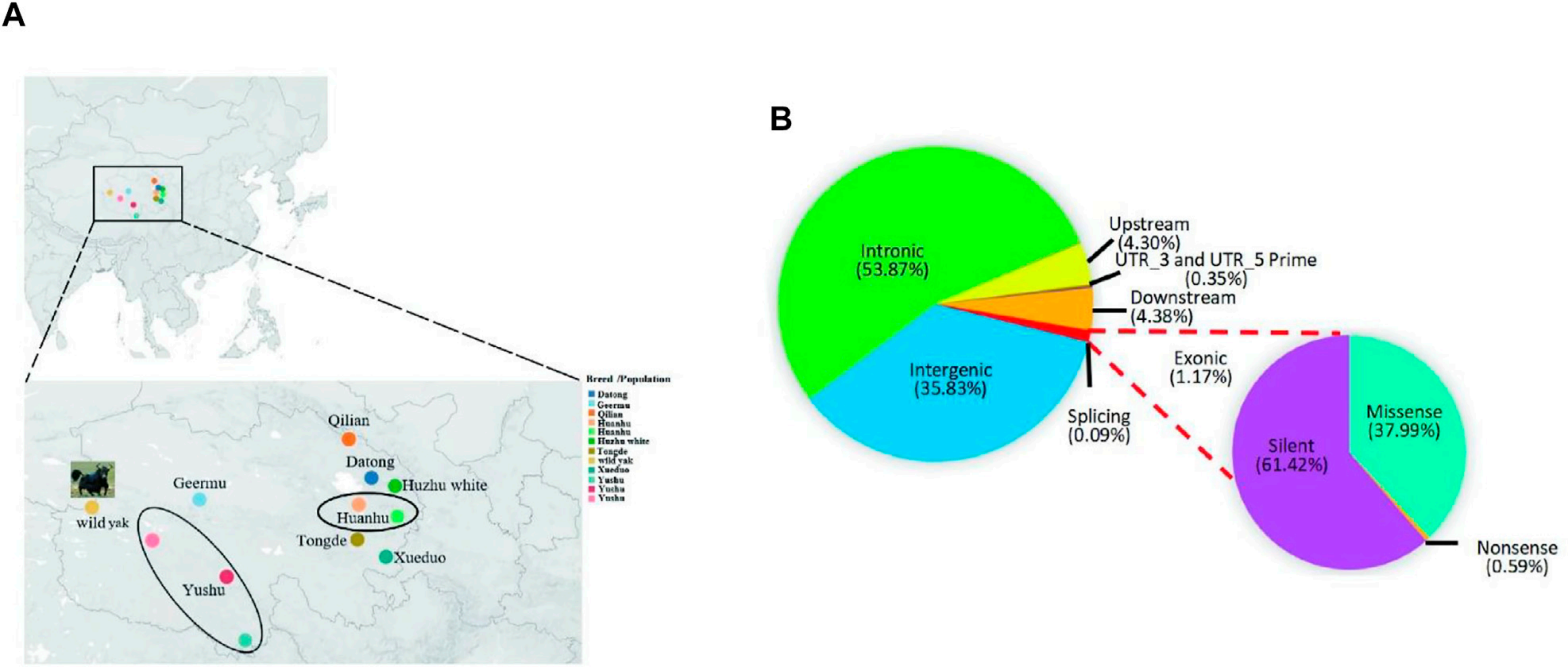
Distribution of samples of Qinghai yak breeds/populations and SNPs in yak genome. **(A)** Sampling sites and geographic distributions of nine Qinghai yak breeds/populations. **(B)** Distribution of detected SNPs in the genomes of nine yak breeds/populations. The circles represent the sampling ranges of Huanhu and Yushu yak.

Based on our previous report on Y-chromosome variations in Qinghai yak (Ma et al., 2018), 42 individuals (NCBI: PRJNA827919) representing breed/population-specific haplotypes were selected for resequencing in this study. Peripheral blood samples were collected from the primary producing area of each Qinghai yak breed/population (Figure 1A; Supplementary Table S1), including Datong (DT, *n* = 6), Yushu (YS, *n* = 6), Xueduo (XD, *n* = 5), Huanhu (HH, *n* = 5), Geermu (GEM, *n* = 5), Qilian (QL, *n* = 5), Tongde (TD, *n* = 7), and Huzhu white (HZ, *n* = 3). Genomic DNA was extracted using the Whole Blood DNA Extraction Kit (Aidlab Biotechnologies Co., Ltd, China) (Supplementary Table S2). Pair-end libraries were constructed for each individual (500 bp insert size), and the quantified DNA was subjected to the Illumina Nova 6000 sequencing platform using a 2 × 150 bp model at the Novogene Bioinformatics Institute (Beijing, China) (Supplementary Table S3). To systematically explore the genomic diversity, population structure, and selection signature of Qinghai yak breeds/populations, we conducted a comprehensive search for whole Qinghai yak genome sequences in the NCBI database, resulting in 71 whole-genome sequences of six Qinghai yak breeds/populations for combined analysis, including wild (WY, *n* = 19), Datong (DT, *n* = 23), Yushu (YS, *n* = 16), Huanhu (HH, *n* = 7), Geermu (GEM, *n* = 3), and Qilian (QL, *n* = 3). In total, this study analyzed 113 whole-genome sequences from nine Qinghai yak breeds/populations (Supplementary Table S1).

### Read mapping and single-nucleotide polymorphism calling

The raw reads were trimmed using Trimmomatic (sliding window: 3:15 minlen:35 trailing:20 leading:20 avgqual:20 tophred33) (Bolger et al., 2014) to remove adapters and low-quality bases. The clean reads were aligned to the latest yak reference genome (BosGru3.0, GCA_005887515.2) using Burrows–Wheeler Aligner (v0.7.13-r1126) software (Li and Durbin, 2009) with default parameters. Picard tools (http://broadinstitute.github.io/picard) were used to filter potential duplicate reads. We used the “Haplotype Caller,” “Genotype GVCFs,” and “Select Variants” modules of the Genome Analysis Toolkit (GATK, v3.8-1-0-gf15c1c3ef) (McKenna et al., 2010) to call the SNPs. After SNP calling, we used the “Variant Filtration” module of GATK to obtain high-quality SNPs with the parameters (“DP <249 (1/3-fold total sequence depth for all individuals) || DP >2245 (three-fold of total sequence depth for all individuals) || QD <2.0 || FS >60.0 || MQ <40.0 || MQRankSum <−12.5 || ReadPosRankSum <−8.0 || SOR >3.0”). Finally, based on the yak reference genome (BosGru3.0, GCA_005887515.2), SNPs were functionally annotated by ANNOVAR software (Wang et al., 2010). In addition, the SNP densities of each Qinghai yak breed/population were calculated by VCFtools (v0.1.12) (window 100,000) software (Danecek et al., 2011).

### Population genomic parameters

To reveal the genomic variations of Qinghai yak, we calculated the genomic parameters, including nucleotide diversity (Pi), expected heterozygosity (He), observed heterozygosity (Ho), inbreeding coefficient (*F*_Hom_), linkage disequilibrium (LD) decay, and runs of homozygosity (ROH) for nine Qinghai yak breeds/populations (Datong, Geermu, Huanhu, Huzhu white, Qilian, Tongde, Xueduo, Yushu, and wild yak).

We estimated the genomic nucleotide diversity of each yak breed/population using VCFtools (v0.1.12) software (-window-pi 50,000 -window-pi-step 20,000). The output of the -het function by VCF tools was a summary for each individual of the observed number of homozygous sites [O(hom)] and the expected number of homozygous sites [E(hom)]. It also included the total number of sites for which the individual had data and the inbreeding coefficient (*F*_Hom_), which was the canonical estimate of genomic *F*_Hom_ based on excess SNP homozygosity (Keller et al., 2011). The SNPs of 113 genomes of nine Qinghai yak breeds/populations were pruned at high levels of pairwise LD by PLINK (v1.9) software (Purcell et al., 2007), excluding SNPs in strong LD (*r* ^2^ > 0.2) within a sliding window of 50 SNPs advanced by five SNPs at a time (Zhang et al., 2019).

The ROHs were calculated by PLINK (v1.9) software. SNPs with minor allele frequencies (MAF < 0.05) were excluded due to instability. PLINK (v1.9) software was used for sliding windows of a minimum of 50 SNPs across the genomes to identify ROHs, allowing for two missing SNPs and one heterozygous site per window. The minimum number of continuous homozygous SNPs constituting an ROH was set to 100. The minimum SNP density coverage was set to at least 50 SNPs per kb, allowing centromeric and SNP-poor regions to be algorithmically excluded from the analysis. The maximum gap between two consecutive homozygous SNPs was set at 100 kb. The number and length of ROHs for each yak breed/population were estimated, and the length of ROHs was divided into three categories: 0.5–1 Mb, 1–2 Mb, and > 2 Mb, reflecting ancient, historical, and recent inbreeding, respectively (Kirin et al., 2010; Bhati et al., 2020).

### Population genetic structure and clustering pattern

VCFtools (v0.1.12) software was used to convert the VCF files into the PLINK format. Linkage sites in the genomic data were removed with parameters (-indep-pair-wise 50 5 0.2) using PLINK (v1.9) software, and then the filtered data were used for principal component analysis (PCA) and admixture analysis. The population genetic structure was estimated using ADMIXTURE (v1.3.0) (Alexander and Lange, 2011), considering 2–4 clusters (*K*), and the results were visualized *via* R (v3.6.1) software. Based on the pairwise distance matrix among individuals, a neighbor-joining (NJ) tree was constructed by MEGA (v10.2.6) (Saitou and Nei, 1987; Kumar et al., 2016). The PCA of 113 individuals was performed by smartPCA in the EIGENSOFT (v5.0) package (Patterson et al., 2006) to estimate the eigenvectors. The Tracy–Widom distribution was used to assess the significance of each principal component, and the results of the first and second principal components were plotted using the ggplot2 package in R (v3.6.1) software. VCFtools (Danecek and McCarthy, 2017) software was used to calculate the fixation index (*F*_ST_) between nine Qinghai yak breeds/populations, and the results were visualized *via* ImageGP (http://www.ehbio.com/ImageGP/). To further explore the relationships among nine Qinghai yak breeds/populations, we calculated the linearized *R*_ST_ values (*R*_ST_ = *F*_ST_/(1- *F*_ST_)). Based on the *R*_ST_ values, the cluster pattern among nine Qinghai yak breeds/populations was revealed by multidimensional scale (MDS) analysis using SPSS (v18.0) software.

### Genome-wide selective sweep

Only SNPs with less than 10% missing nucleotides were used for selective sweep scanning. To explore the selection pressure of domestic and wild yak in Qinghai, we performed a detection of positive selection signature. In this study, three methods, including composite likelihood ratio (CLR), nucleotide diversity (PI), and pairwise fixation index (*F*_ST_), were used to detect the selection signatures in Qinghai yak. The CLR was computed using SWEEPFINDER2 software to detect SNPs within a non-overlapping 50 kb window (Nielsen et al., 2005; DeGiorgio et al., 2016). The PI was estimated using VCFtools (50 kb sliding window and 20 kb step) in Qinghai domestic yak. Consistent with previous methods, the top 1% windows were selected as the candidate region under selection (Chen et al., 2018; Xia et al., 2021). To find candidate genes related to adaptation, immunity, and economic traits, the *F*_ST_ was calculated between wild and domestic yak with a 50-kb sliding window and 20-kb steps along the autosomes using the VCFtools and R scripts (Danecek et al., 2011; Chen et al., 2018; Chen et al., 2020; Xia et al., 2021). The Tajima’D values were calculated for the candidate genes using VCFtools. To gain a further understanding of the gene functions and signaling pathways of the identified candidate genes, online Kyoto Encyclopedia of Genes and Genomes (KEGG) pathway and Gene Ontology (GO) analyses were conducted using KOBAS 3.0 (http://kobas.cbi.pku.edu.cn/anno_iden.php), and FDR < 0.05 was used as a threshold to detect significantly enriched genes and pathways.

## Results

### Whole-genome resequencing and single nucleotide polymorphisms

The total raw and clean bases of 42 newly sequenced Qinghai yak were 3,974.07 Gb and 3,942.42 Gb, respectively (Supplementary Table S3). The average sequencing depth of the reads in 42 individual genomes was around ×23.91 (Supplementary Table S1). In total, we obtained 112,960,043 high-quality SNPs in nine yak breeds/populations. The highest number of SNPs (18,061,823) was detected in Datong, followed by Yushu (14,833,892), wild (14,109,444), Huanhu (12,805,435), Tongde (12,059,879), Qilian (11,462,122), Xueduo (10,394,482), and Geermu (10,266,014) yak, while the lowest SNPs were detected in Huzhu white yak (8,966,952) (Supplementary Table S4). Comparison among 113 yak genomes revealed that, among 25,768,146 autosomal SNPs, 35.83% were mapped to intergenic regions, 53.87% to intronic regions, and only 1.17% to exonic regions. The functional annotation of the SNPs assigned to protein-coding regions identified about 61.42% of SNPs to produce silent mutations, 37.99% to cause missense mutations, and 0.59% to result in nonsense mutations (Figure 1B; Supplementary Table S4).

### Population genomic diversity, runs of homozygosity, and linkage disequilibrium

**FIGURE 2:**
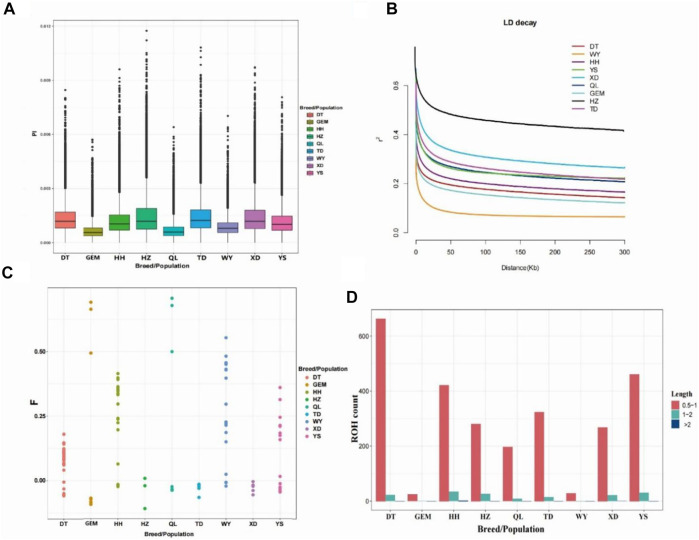
Analyses of genomic variation, LD, *F*_Hom_, and ROHs for Qinghai yak breeds/populations. **(A)** Box plots of nucleotide diversity (Pi) for each yak breed/population. **(B)** Decay of LD in nine yak breeds/populations, with one line per breed/population. **(C)** *F*_Hom_ in nine yak breeds/populations. **(D)** Length and average number of ROHs in nine yak breeds/populations.

Nucleotide diversity (Pi) ranged from 0.0006 to 0.0015 among nine Qinghai yak breeds/populations (Figure 2A; Table 1), with the highest Pi value (0.0015) in Huzhu white yak and the lowest (0.0006) in Geermu yak (Figure 2A; Table 1). The values of He and Ho ranged from 0.2699 to 0.4752 and from 0.1443 to 0.3454 for Datong and Huzhu white yak, from lowest to highest, respectively (Table 1). LD analysis showed that the wild yak presented a very rapid decay rate and the lowest level of LD (Figure 2B). However, in the domestic yak breeds/populations, the Huzhu white yak exhibited a slow decay rate and a high level of LD, followed by Xueduo, Tongde, Yushu, Qilian, Huanhu, and Datong yak, whereas the Geermu yak showed a rapid decay rate and a low level of LD. Among nine Qinghai yak breeds/populations, the wild yak had the highest value of *F*_Hom_ (0.2841), followed by Qilian, Yushu, Geermu, Huanhu, Datong, Xueduo, Tongde, and Huzhu white yak (Table 1; Figure 2C). We found that the ROH lengths in all yak breeds/populations were mostly between 0.5 and 2 Mb (Figure 2D; Supplementary Table S5). Larger ROHs (>2 Mb) were only identified in Huanhu (2) and Xueduo (1) yak, while medium ROHs (1–2 Mb) were found in almost all the yak breeds/populations, except for the wild yak (i.e., Datong (22), Geermu (1), Huanhu (34), Huzhu white (26), Qilian (9), Tongde (14), Xueduo (21), and Yushu (30)) (Figure 2D; Supplementary Table S5).

**TABLE 1:** Genetic diversity indexes of nine yak breeds/populations in Qinghai, China.

Breed/population	No. of sample/sequence	Pi	Ho	He	Fhom
Datong yak	29	0.0013	0.1443	0.2699	0.0687
Geermu yak	8	0.0006	0.2677	0.2835	0.1817
Huanhu yak	12	0.0012	0.2283	0.3879	0.1747
Huzhu white yak	3	0.0015	0.3454	0.4752	−0.0394
Qilian yak	8	0.0007	0.2429	0.4461	0.2153
Tongde yak	7	0.0014	0.2457	0.2881	−0.0271
Xueduo yak	5	0.0014	0.2932	0.3747	−0.0269
Yushu yak	22	0.0011	0.1892	0.2961	0.2039
Wild yak	19	0.0009	0.2017	0.2798	0.2841

Note: Pi, nucleotide diversity; Ho, observed heterozygosity; He, expected heterozygosity; Fhom, excess of homozygosity inbreeding coefficient.

### Population genetic structure

**FIGURE 3:**
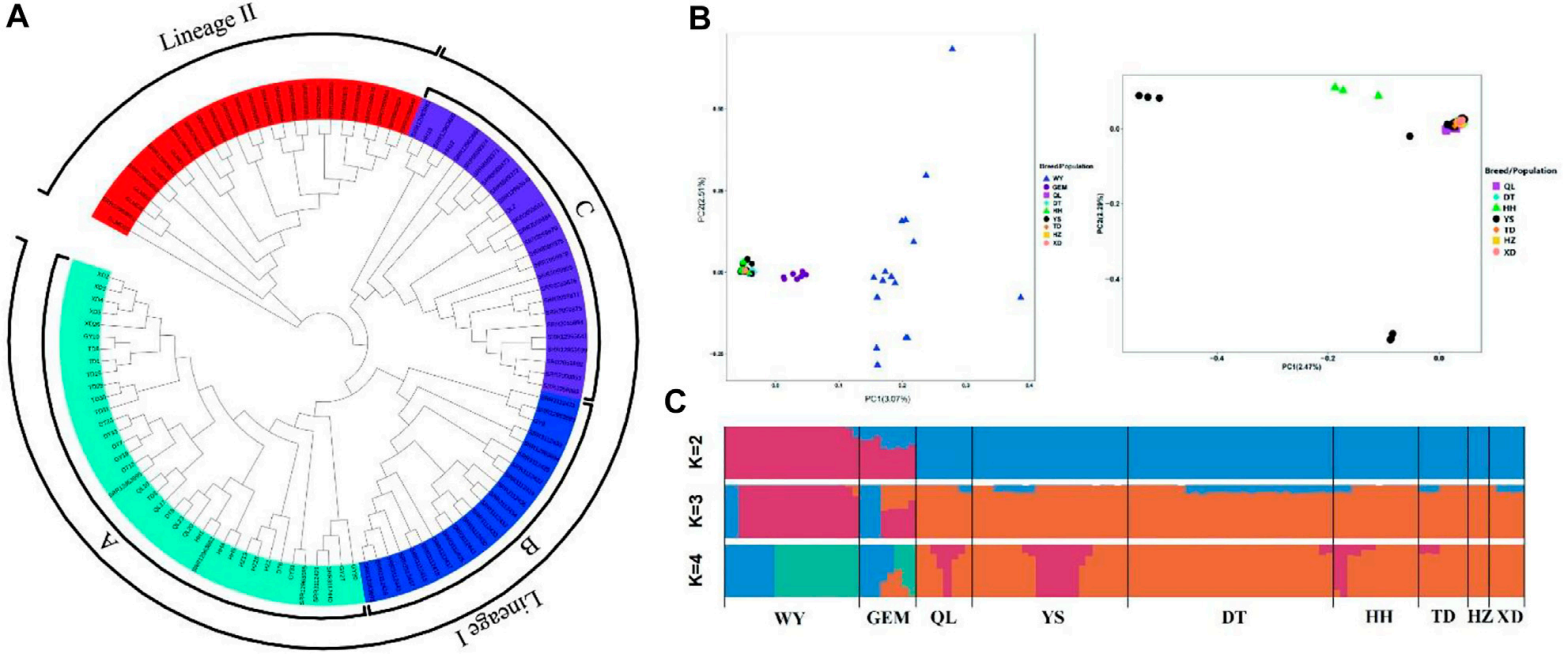
Population genetic analyses of Qinghai yak breeds/populations. **(A)** NJ tree of 113 wild and domestic yak. **(B)** PCA with first (PC1) and second (PC2) principal components. The right figure shows a further PCA excluding wild and Geermu yak populations. **(C)** Model-based clustering of Qinghai yak breeds/populations using ADMIXTURE with *K* = 2–4.

To determine the population genetic structure and relationships among nine different yak breeds/populations, we conducted a series of analyses, including phylogenetic reconstructions, PCA, and Bayesian clustering, using WGS data. In a phylogenetic analysis, the NJ tree showed different clades for wild yak and eight domestic yak breeds/populations in Qinghai and inferred two lineages (lineages I and II) (Figure 3A) and three sub-lineages (A, B, and C) in Lineage I.

According to the PCA results, the first principal component (PC1) explained 3.07% of the total variation separating wild yak and Geermu yak from the other seven yak breeds/populations (Figure 3B). The second PC (PC2) explained 2.51% of the total variation, indicating a more complex genetic makeup in wild yak than in other Qinghai domestic yak breeds/populations. In addition, a further PCA analysis excluding the wild and Geermu yak populations showed that some individuals from Yushu and Huanhu yak were divided (Figure 3B). To further elucidate the population genetic structure of Qinghai yak breeds/populations, we estimated the ancestral components for 113 individuals *via* ADMIXTURE software through clustering models (Figure 3C). In clustering analysis, when *K* = 2, wild and Qinghai domestic yak breeds/populations had two ancestral components, namely, wild yak and domestic yak, with only the Geermu yak presenting two ancestral components. When *K* = 3, a new ancestry was observed in almost all Qinghai yak breeds/populations except Huzhu white yak. Notably, the wild yak and Geermu yak populations accounted for a relatively high proportion of this new ancestry, but the other six Qinghai domestic yak breeds/populations (Qilian, Yushu, Datong, Huanhu, Tongde, and Xueduo) shared a small amount of this new ancestry. When *K* ≥ 4, cross-validation errors (≥0.3043) for ancestry models increased gradually, which suggested false results for the ancestry analysis (Supplementary Table S13).

**FIGURE 4:**
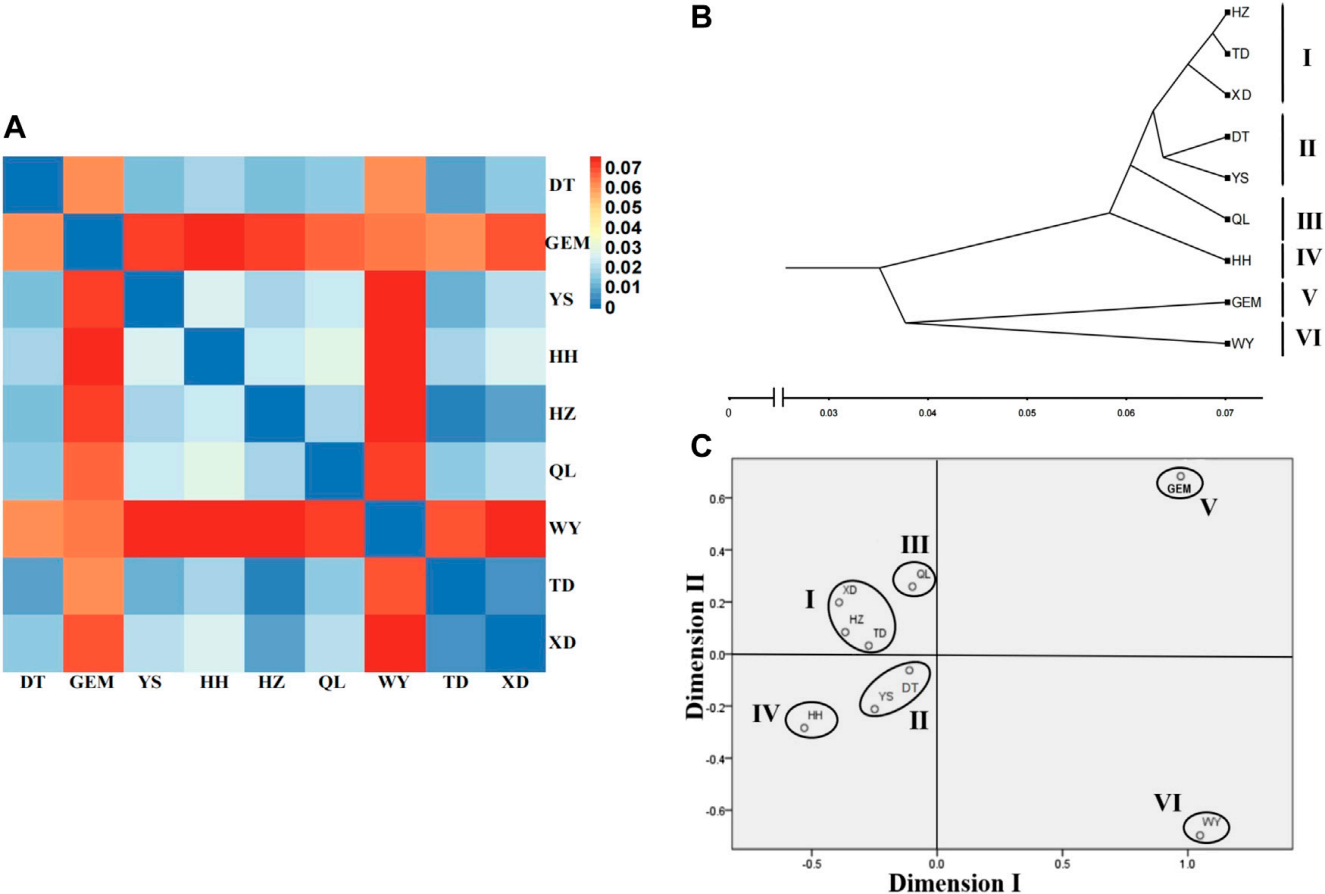
Genetic relationships among Qinghai yak breeds/populations. **(A)** Heat map of genetic differentiation among nine yak breeds/populations. **(B)** Cluster relationship among nine yak breeds/populations using the UPGMA tree. **(C)** MDS analysis among nine yak breeds/populations.

The fixation index (*F*_ST_) values between nine yak breeds/populations ranged from 0.003 to 0.076 (Supplementary Table S6), which showed moderate genetic differentiation (0.05 < *F*_ST_ < 0.15) between wild yak, Geermu yak, and the other seven yak breeds/populations. However, there was weak genetic differentiation (0 < *F*_ST_ < 0.05) among the other seven yak breeds/populations (Figure 4A, Supplementary Table S6). Based on the *R*_ST_ values (Supplementary Table S6), the clustering analysis showed that nine yak breeds/populations were separated into six groups (groups I–VI) (i.e., wild; Geermu; Huanhu; Qilian; Yushu and Datong; and Xueduo, Tongde, and Huzhu) (Figures 4B,C).

### Genome-wide selective sweeps

**FIGURE 5:**
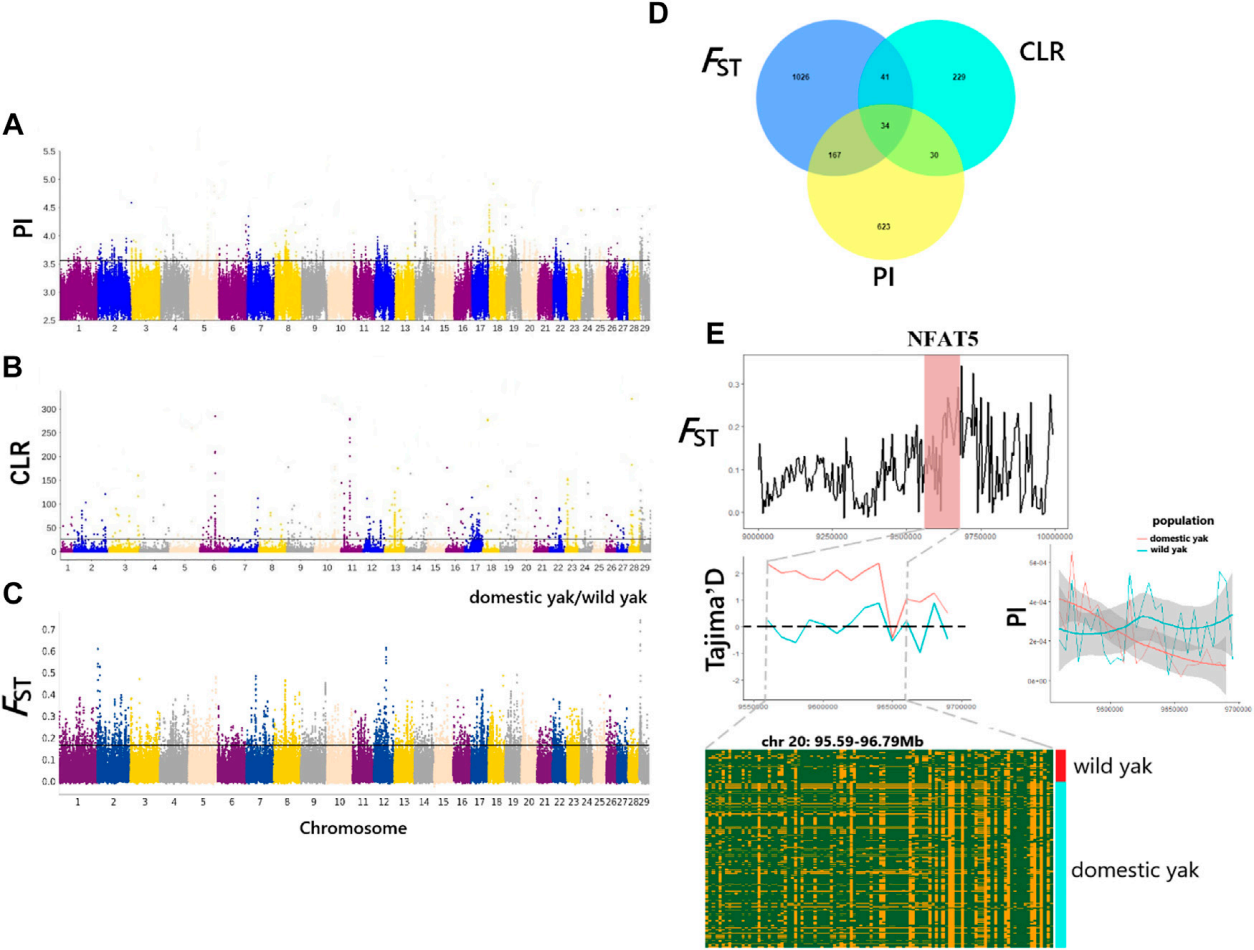
Genome-wide selection scan for positively selected genes (PSGs) in Qinghai yak using sliding window analysis (50-kb window size and 20-kb step size). **(A)** Manhattan plot of selected sweeps by the PI method. **(B)** Manhattan plot of selected sweeps by the CLR method. **(C)** Manhattan plot of selected sweeps by the *F*_ST_ method, based on domestic and wild yak populations. **(D)** Venn diagram showing the candidate genes identified by the CLR, PI, and *F*_ST_ methods. **(E)** *F*_ST_, Tajima’D, and PI of the *NFAT5* gene region between wild and domestic yak.

In this study, we used the CLR, PI, and *F*_ST_ methods to screen for the potentially selected genomic regions in wild yak and eight domestic yak breeds/populations in Qinghai (Figures 5A–C). As a result, a total of 334 (CLR), 854 (PI), and 1268 (*F*_ST_) genes were identified (Supplementary Tables S7–S9), with only 34 genes overlapping among the three analyses (*p* < 0.05) (Supplementary Tables S10, Figure 5D). These identified genes were considered candidate positively selected genes (PSGs) (Figures 5A–C). Annotations of the 34 candidate genes revealed functions that may be associated with important traits, including disease resistance (*CDK2AP2*, *PLEC*, and *CYB5B*), heat stress (*NFAT5*, *HSF1* and *SLC25A48*), pigmentation (*MCAM*, *RNF26*, and *BOP1*), vision (*C1QTNF5*, *MFRP*, and *TAX1BP3*), milk quality (*OPLAH* and *GRINA*), neurodevelopment (*SUSD4*, *INSYN1*, and *PPP1CA*), and meat quality (*ZRANB1*) (Supplementary Tables S10). As a suppressor of oxidative phosphorylation-associated gene expression, mitochondrial respiration, and reactive oxygen species (ROS) production in pulmonary artery smooth muscle cells, *NFAT5* gene is vital in limiting ROS-dependent arterial resistance in hypoxic environments (Laban et al., 2021). Here, it is notable that the genomic region harboring *NFAT5* (95.59–96.79 Mb on chromosome 20) exhibited higher *F*_ST_ and differential Tajima’D and PI values between wild and domestic yak (Figure 5E). However, the haplotype diagram showed that differences in this gene region between wild and domestic yak were not obvious. To further elucidate the genetic mechanisms related to the candidate genes, we also performed functional enrichment analysis for the 34 candidate genes, using KOBAS (http://kobas.cbi.pku.edu.cn/) to find vital KEGG and GO pathways. In total, 29 KEGG pathways and 383 GO terms were found in our enrichment results (Supplementary Tables S11, S12). As for functional enrichment analysis (Supplementary Table S11), the KEGG pathway had four significant functions, called “regulation of actin cytoskeleton,” “glycosylphosphatidylinositol (GPI)-anchor biosynthesis,” “Legionellosis”, and “glutathione metabolism” (*p*-value < 0.05), as well as five genes (*ITGAE*, *PPP1CA*, *GPAA1*, *HSF1*, and *OPLAH*) related to disease resistance, growth, and heat stress (Supplementary Table S10). GO terms were particularly enriched in terms of osmotic stress and endoplasmic reticulum stress (“response to osmotic stress, GO:0006970,” “nuclear stress granule, GO:0097165,” and “negative regulation of endoplasmic reticulum stress-induced intrinsic apoptotic signaling pathway, GO:1902236”). Several genes (*NFAT5*, *MARVELD3*, *HSF1*, and *GRINA*) were found to be associated with immunity, heat stress, and milk quality. We also detected significant GO terms responsible for growth (“cellular response to platelet-derived growth factor stimulus, GO:0036120,” “positive regulation of multicellular organism growth, GO:0040018,” and “cellular response to transforming growth factor beta stimulus, GO:0071560”) (Supplementary Table S12) involving relevant genes (*CORO1B*, *HSF1*, and *SCX*). In addition, some significant GO terms (“protein binding, GO:0005515,” “acyl carnitine transmembrane transporter activity, GO:0015227,” and “acyl carnitine transmembrane transport, GO:1902616”) related to disease resistance were detected, which may contribute to disease resistance of yak in harsh high-altitude environments.

## Discussion

Genetic diversity is an important component of biological diversity. It is the basis of biological evolution and species differentiation, with great significance for population maintenance and adaptation to habitat change. In a previous study, Ji et al. (2021) suggested that wild yak had experienced a genetic bottleneck, yet that their genomic diversity was still higher than that of domestic yak. However, Qiu et al. (2015) concluded that the value of genomic nucleotide diversity in domestic yak (0.0014) was slightly higher than that of wild yak (0.0013). Chai et al. (2020) also showed that the genomic diversity of wild yak (0.0012) was lower than that of some domestic yak breeds/populations (0.0010–0.0016). In this study, the values of nucleotide diversity for nine Qinghai yak breeds/populations ranged from 0.0006 to 0.0015 at the whole-genome level (Table 1; Figure 2A), which indicates a wide range of genomic diversity for wild yak and eight Qinghai domestic yak breeds/populations. Here, the wild yak had nucleotide diversity of 0.0009, Huzhu white yak had the highest nucleotide diversity (0.0015), and Geermu yak had the lowest nucleotide diversity (0.0006). The nucleotide diversities of most of domestic yak breeds/populations, except for Geermu and Qilian yak, were higher than that of wild yak, indicating that most of Qinghai yak breeds/populations had abundant genomic diversity; our result is consistent with previous reports (Table 1; Figure 2A) (Qiu et al., 2015; Chai et al., 2020).

ROH has gradually become an important index for identifying inbreeding degrees and genetic variation patterns in livestock populations (Chen et al., 2018; Xia et al., 2021). Here, the ROH distribution pattern of nine yak breeds/populations showed significant differences (Figure 2D, Supplementary Table S5). The ROH numbers for Geermu and wild yak populations were small, which indicates that the living habitats of these yak populations were remote from human settlements and had less artificial intervention. Among the other domestic yak breeds/populations, Datong yak had the highest ROHs in both length and number (Figure 2D, Supplementery Table S5), followed by Yushu, Huanhu, Tongde, Huzhu white, Xueduo, and Qilian yak. The values indicated that these seven yak breeds/populations have been subjected to significant human intervention. In addition, the pattern of LD decay in each yak breed/population was largely consistent with the results of nucleotide diversity in this study. The results of inbreeding coefficients inferred that Geermu, Huanhu, Qilian, Yushu, and wild yak breeds/populations had high degrees of inbreeding (Figures 2B,C; Table 1). It is noteworthy that high selection pressure may cause inbreeding depression in yak.

In previous studies, Qiu et al. (2015) detected a clear genetic split between wild and domestic yak despite low morphological divergence and continuing gene flow between them. Similarly, Chai et al. (2020) noted that when *K* = 2, yak populations were divided into domestic and wild yak, while when *K* = 3–5, yak samples could not be divided into different ancestries, which indicates extensive genetic mixing among domestic yak. In our present study, we explored the population genetic structure of wild yak and Qinghai domestic yak breeds/populations. The ADMIXTURE analysis showed that at *K* = 2, nine yak breeds/populations had two ancestral components (domestic yak and wild yak), while Geermu yak carried a high genetic component of wild yak. At *K* = 3, a third new ancestral component appeared in a few wild yak individuals and in all domestic yak breeds/populations except for Huzhu white yak. Geermu yak showed more similar ancestral composition to wild yak, and other domestic yak breeds/populations in Qinghai showed similar ancestral components that differed from wild yak. The size of cross-validation errors could affect the authenticity of ancestry (Chen et al., 2018; Xia et al., 2021). At *K* = 2, the minimum CV value (0.2878, Supplementary Table S12) indicated the accurate ancestral composition of Qinghai yak, and the obtained result was consistent with previous studies (Qiu et al., 2015; Chai et al., 2020). According to PCA, among 113 individuals of nine breeds/populations in Qinghai, the Qinghai yak were divided into three clusters: wild yak, Geermu yak, and other domestic yak breeds/populations (Datong, Huanhu, Yushu, Qilian, Xueduo, Tongde, and Huzhu white yak) (Figure 3B). In an early PCA, separation among domestic yak samples demonstrated that all domestic yak populations showed a single-origin domestication and close genetic distance (Chai et al., 2020), which is similar to our current findings.

*F*_ST_ is the index of genetic differentiation among populations and is used to evaluate the degree of differentiation among populations. *F*_ST_ may show very weak (0–0.05), moderate (0.05–0.15), or significant (0.15–0.25) population differentiation. The degree of differentiation is considered extremely significant when the index exceeds 0.25 (Wright, 1978). In a previous study, based on Y-SNPs and Y-STR markers, the average *F*_ST_ value between wild yak and 15 domestic yak populations was 0.178, indicating significant paternal genetic differentiation between domestic and wild yak (Ma, 2019). In the present study, the *F*_ST_ values between nine yak breeds/populations were 0.003–0.076 (Figure 4A, Supplementary Table S6), which suggests moderate genetic differentiation between wild yak, Geermu yak, and the seven other yak breeds/populations, and weak genetic differentiation among the other seven yak breeds/populations. The result confirmed that the Geermu yak population has certain hereditary particularities. Nowadays, Datong, Huanhu, Xueduo, and Yushu yak in Qinghai have been recognized as different breeds by China National Committee of Animal Genetic Resources (National Committee of Animal Genetic Resources, 2021). In our MDS analysis (Figures 4B,C), nine yak breeds/populations were divided into six groups (I–VI) (wild; Geermu; Huanhu; Qilian; Yushu, and Datong yak; and Xueduo, Tongde, and Huzhu white yak). Here, Geermu and Qilian yak populations had significant genetic differences from other yak breeds/populations, which therefore could be considered as potential new genetic resources for further research and utilization. Notably, the clustering results among yak breeds/populations were not closely related to their geographical distributions, consistent with our PCA and ADMIXTURE analysis results (Figures 3B,C). Previously, studies based on Y-chromosome marker variations showed that both wild and domestic yak in Qinghai had relatively high paternal genetic diversity with weak phylogeographic structure and two paternal origins (Ma et al., 2018; Ma, 2019). Maternal genetic diversity in wild and domestic yak breeds/populations (Qinghai-Plateau, Huanhu, Xueduo, and Yushu yak) in Qinghai was recently examined based on nucleotide variants of mitogenomes, and the results suggested that both domestic and wild yak from Qinghai contain a wide range of maternal variability; the genetic differentiation among Qinghai indigenous yak was weak, but each Qinghai indigenous yak breed had unique maternal genetic information (Li GZ et al., 2022). In the phylogeny of yak maternal origins, the wild yak and Qinghai domestic yak were composed of three maternal lineages (lineages Ⅰ, Ⅱ, and Ⅲ) with three possible maternal origins, but only a few wild and domestic yak carried lineage III (Li et al., 2022). In this study, the clustering of nine yak breeds/populations into two large lineages (lineages Ⅰ and Ⅱ) suggests that Qinghai yak might have two origins. Among them, three sub-lineages (A, B, and C) were determined to belong to lineage Ⅰ, and no sub-lineage was found in lineage II. This result is not consistent with previous results of yak maternal origin studies but is similar to results of yak paternal phylogenetic analysis (Ma, 2019; Li et al., 2022). We acknowledge that our population genetic structure results are sensitive to the relatively limited sample sizes, but they do roughly reflect the genetic background of Qinghai yak breeds/populations included in this study.

The identification of genomic signatures of selection helps reveal genetic mechanisms underlying traits of importance in yak. Overall, 34 PSGs identified in nine Qinghai yak breeds/populations using three integrated methods appear to be possibly related to important traits in humans or other animals. For example, *NFAT5*, a member of the NFAT gene family related to immunity in humans (Dalski et al., 2001), was identified as a PSG in Qinghai yak. Therefore, *NFAT5* gene might be related to the adaptation of Qinghai yak to extreme environments and to their evolution of a powerful mitochondrial function to resist hunger, hypoxia, and severe cold. It is noticeable that the region scanned by *F*_ST_ for *NFAT5* exhibited higher *F*_ST_, and differential Tajima’D and PI values, between wild and domestic yak, indicating strong selective sweeps. Furthermore, a diagram of the haplotypes shows that this gene region is not stable in domestic yak. The heat shock factor 1 (*HSF1*) gene is a regulator of the heat stress response, maximizing heat shock protein expression, and thus is certified to be associated with heat tolerance in Jersey, Angus, Simmental, and indicine cattle (de Fátima Bretanha Rocha et al., 2019; Rong et al., 2019; Mohapatra et al., 2021). Here, it was identified as a yak PSG, and we therefore speculated that this gene is related to yak’s resistance to high-intensity ultraviolet rays and frigid climates on the QTP. In addition, the CNV of *HSF1* gene was shown to be related to growth and development in Ashidan yak (Ren et al., 2022). However, further research on this vital PSG in yak is needed. The association of economic traits with candidate genes has revealed that *ZRANB1* polymorphisms are related to the muscle pH, conductivity, flesh color, and drip loss in pigs (Huynh et al., 2013). Thus, we speculated that SNPs in this gene might be the causal sites that affect water holding capacity (WHC) in yak meat. Notably, based on the three selected methods, two genes (*GRINA* and *OPLAH*) linked to milk yield and fat contents in Holstein cows (Atashi et al., 2020; Peters et al., 2021) also showed positive selection, which may be related to milk traits in Qinghai yak.

Coat color is an important target of selection in many domestic animals. Black coat color plays an important role in protecting yak from ultraviolet radiation on the QTP. Previously, *MCAM* and *RNF26* were considered key determinants of white/black tail feather color in dwarf chickens (Nie et al., 2021). Additionally, Bao et al. (2020) revealed some modular hub genes highly related to hormones such as *SLF2*, *BOP1,* and *DPP8*, which are involved in hormone regulation related to the hair cycle in yak. In this study, three genes (*MCAM*, *RNF26*, and *BOP1*) were identified as PSGs by all three methods. We believe that these three genes are more likely to participate in hair formation and pigmentation in yak, although additional functional experiments are needed for verification. Additionally, *SUSD4*, a gene coding for a complement-related transmembrane protein involved in neurodevelopment (González-Calvo et al., 2021), may participate in cold adaptation in Qinghai yak. Overall, our study reveals several PSGs in Qinghai yak, contributing to an improved understanding of the genetic mechanisms of population characteristics and providing a molecular basis for yak breeding.

## Conclusion

In conclusion, this study provides a comprehensive overview of the genomic diversity, population structure, and selection signatures of Qinghai yak using whole-genome resequencing data. Overall, most Qinghai yak breeds/populations possess abundant genomic diversity. The Qinghai yak have two ancestral components (domestic and wild yak), and the Geermu yak carry a high genetic component originating from wild yak. Moreover, Qinghai yak are clustered into wild, Geermu, and other seven yak breeds/populations, while weak genetic differentiation is displayed among the seven other domestic yak breeds/populations. The candidate genomic regions are involved in disease resistance, heat stress, pigmentation, vision, milk quality, neurodevelopment, and meat quality. This research indicates for the first time that Geermu yak, Qilian yak, and the other domestic yak breeds/populations exhibit genetic differences at the genomic level. These findings provide a theoretical basis for the reasonable protection and utilization of Qinghai yak genetic resources in the future.

## Data Availability

The datasets presented in this study can be found in online repositories. The data presented in the study are deposited in the NCBI repository, accession BioProject number: PRJNA827919.
